# Improving Men’s Participation in Preventing Mother-to-Child Transmission of HIV as a Maternal, Neonatal, and Child Health Priority in South Africa

**DOI:** 10.1371/journal.pmed.1001811

**Published:** 2015-04-07

**Authors:** Wessel van den Berg, Kirsty Brittain, Gareth Mercer, Dean Peacock, Kathryn Stinson, Hanna Janson, Vuyiseka Dubula

**Affiliations:** 1 Sonke Gender Justice, Cape Town, South Africa; 2 School of Public Health & Family Medicine, University of Cape Town, Cape Town, South Africa; 3 School of Population and Public Health, University of British Columbia, Vancouver, Canada; 4 Vaccine Evaluation Centre, Department of Pediatrics, British Columbia Children’s Hospital, Vancouver, Canada; 5 Médecins Sans Frontières, Khayelitsha, Cape Town, South Africa

## Abstract

Wessel van den Berg and colleagues outline how increasing male partner involvement in efforts to reduce mother-to-child HIV transmission in South Africa may improve maternal and infant outcomes.

**Summary Points**

- Involving male partners in programmes to prevent mother-to-child transmission of HIV may improve programme coverage and infant outcomes.
- Rates of male partner involvement remain low worldwide, and detailed guidelines to increase involvement are lacking in South Africa.
- We recommend that South African national and provincial guidelines and policies for preventing mother-to-child HIV transmission be adjusted to explicitly include a focus on increasing male partner involvement and that they include concrete descriptions of how to achieve this.
- We propose recommendations for improving male partner involvement at a policy, facility, and community level.
- Challenges to improving male partner involvement include the nature of relationships and family structures in South Africa and the capacity of health systems to implement recommendations.

## Introduction

The World Health Organization promotes a four-component strategy for preventing mother-to-child transmission (PMTCT) of HIV: prevent new infections in women; prevent unintended pregnancies among women living with HIV infection; prevent transmission of HIV from mothers to their children during pregnancy and breastfeeding; and identify, treat, and support women living with HIV, their children, and their families [1].

Historically, PMTCT services have been directed primarily at women. Ignoring men’s influence on reproductive health has probably limited both the reach and the effectiveness of these services. Strategies to increase male partner involvement (MPI) in PMTCT programmes are aimed at improving programme results and allowing programmes to reach more clients [2]. Recruiting men as supportive partners in PMTCT can improve the health of women and children, better engage men in their own health, improve the communication of couples, and increase the participation of fathers in child health through increased proximity to the health system and to the child. For these reasons, MPI has been advanced as a priority intervention in PMTCT programmes.

## Evidence for Benefits of Men’s Participation in Preventing Mother-to-Child Transmission of HIV

Studies in sub-Saharan African countries have found that cultural and gender norms give men more power than women in sexual decision-making, including in regards to abstaining, having concurrent sexual partners, and using condoms [3,4]. Such norms may prevent women from accessing family planning and sexual health services unless their partners are also engaged [5]. For HIV-infected mothers, disclosing their HIV status to their partners and being supported in their infant-feeding choices may promote successful exclusive breastfeeding [6,7]. In addition, fear of rejection and violence from partners may be a barrier to HIV testing and returning to the health care facility for test results and antiretroviral drugs [8,9].

Interventions to promote MPI in PMTCT have often involved providing antenatal HIV testing and counselling for couples. Routine HIV testing and early initiation of treatment are highly effective HIV transmission prevention strategies [10]. Targeting couples is promising because it can prevent mother-to-child transmission of HIV, as well as transmission within serodiscordant couples in which the infected partner’s status is unknown. Counselling can improve couples’ communication about HIV, fertility-related decision-making, and gender equality [11].

To date, there have been few rigorous evaluations of PMTCT-based couples counselling and testing interventions, but findings have generally been positive. In sub-Saharan African studies, it was found that women whose partners attended counselling were more likely to accept HIV testing and to adhere to antiretroviral prophylaxis and infant feeding recommendations [12–15]. Women’s disclosure of their HIV status to a male partner has been found to significantly improve antiretroviral adherence during pregnancy in Tanzania [16] and Nigeria [17]. In addition, MPI in PMTCT was shown to decrease infant HIV infection and increase HIV-free survival in a study conducted in Kenya [18]. In the context of PMTCT in South Africa, disclosure and MPI have similarly been shown to improve antiretroviral adherence during pregnancy [19].

## The Policy Dimension of Male Partner Involvement in PMTCT in South Africa

Despite the promise shown in local and international studies, rates of male partner attendance at antenatal care (ANC) visits and participation in couples HIV testing remain low worldwide [20].

The Joint United Nations Programme on HIV and AIDS (UNAIDS) Global Plan argues for the support and involvement of men in PMTCT programmes [21]. Some countries in Africa show commitment to MPI in PMTCT but lack specific guidelines. A recent review of health-related policies of 12 countries [22] in Africa assessed whether the policies contain language on engaging men and boys in areas critical for gender equality. All of the countries noted that men play an important role in PMTCT. Six of the countries (Democratic Republic of Congo, Ethiopia, Malawi, Mozambique, Sierra Leone, and Zimbabwe) were vague in detailing what such male involvement would include. They failed, for example, to mention the care-giving roles men can, or should, play in the lives of their children or to provide detailed plans on how men can provide support to their partners.

In the six remaining countries (Kenya, Namibia, Rwanda, Tanzania, Uganda, and Zambia), the PMTCT policies contained proactive and progressive language. For example, national policies in Tanzania draw attention to the lack of MPI in PMTCT and include detailed strategies for how to engage men. These strategies include developing “male-friendly” spaces in order to encourage men to attend ANC services, aiming for “50% of male partners of women tested for HIV through PMTCT to be screened and counselled for HIV,” and advocating for a change in societal norms. One strategy document outlines potential strategies to address the fact that “the ANC services are not currently organised in a way that encourages joint attendance by both parents” [23].

Male-partner testing rates in southern Africa are well below 50%, and few men attend sexual and reproductive health services with their partners [24]. In a recent study in Cape Town, no men accompanied their partners to antenatal care at the enrolment of mothers in the programme, although 35% did so after the intervention [25]. A study in rural South Africa showed that one man accessed HIV testing services for every three women tested [26].

Reasons for men’s current disengagement from maternal, neonatal, and child health (MNCH) services are complex and longstanding but amenable to change. Important barriers include the persistence of traditional gender norms that discourage men from attending health services (for example, the perception that reproductive health is a “woman’s affair”) and health policies that have focused on women as the only agents for improving maternal and child health and reducing vertical HIV transmission. While these policies have been successful in many ways, they have inadvertently reinforced the exclusion of men and, by extension, couples [20,24,27–29].

The South African National Strategic Plan on HIV, STIs and TB (NSP) mentions the need to strengthen PMTCT programmes by involving and engaging with men [30]. For example, it commits to “engaging women and men, and ensuring that PMTCT is integrated into sexual and reproductive health and fertility management services,” and to strengthening PMTCT services, including through “the engagement of fathers” [31]. However, the importance of engaging with men receives no further attention in the NSP, and there is no discussion concerning how to encourage men to become more involved in PMTCT.

The language of the National Action Framework (NAF) for “No Child Born with HIV by 2015 & Improving the Health and Wellbeing of Mothers, Partners and Babies in South Africa” is more promising. One of the focus areas put forward is the meaningful inclusion and engagement of males in PMTCT services using a family-centered approach. Furthermore, the framework includes an objective of raising awareness and increasing utilisation of services through targeted efforts to reach men and male leaders [30]. It states that strategies should include addressing MPI in training programmes for health care providers in PMTCT, dissemination of materials to enhance male participation, and working with male leaders and role models in the community to advocate for male participation [30]. However, the NAF does not put forward comprehensive recommendations on how to achieve these aims.

The 2010 South African national PMTCT guidelines are even more sparse in their guidance for achieving MPI. One stated aim of the guidelines is to involve women’s partners and families in PMTCT services, yet the words “men,” “man,” “male,” “father,” “parent,” “fatherhood,” and “dad” are conspicuously absent [32]. The same is true for the Province of the Western Cape Department of Health 2013 PMTCT clinical guidelines [33].

Despite the above-mentioned barriers, many studies have demonstrated that it is possible to increase men’s knowledge of sexual and reproductive health, improve MPI in PMTCT, challenge negative attitudes, and increase positive behaviours [34,35]. When given the opportunity, many men wish to be positively involved in reproductive health decision-making, including decisions about the use of services, to contribute to positive health outcomes for themselves and for their families [34,35].

We propose that South African PMTCT services could be enhanced by capitalising on such evidence and acting to reinforce men’s interest in, and shared responsibility for, MNCH outcomes.

## Proposed Policy Adjustment

We recommend that South African National and Provincial PMTCT guidelines and policies include concrete descriptions of how to achieve and measure the following three goals for male partner involvement:

1. **Ensure that men are engaged as supportive partners in MNCH.** A promising approach for achieving this goal is to routinely provide couples with HIV counselling and testing as part of ANC services [36]. Couples HIV testing and counselling should be viewed as a starting point for establishing long-running engagement of men in MNCH services.
  Men’s participation in ANC services could be encouraged by using written invitations [25], offering expanded weekday or weekend clinic hours, or delivering these services directly in clients’ homes [37]. HIV self-testing appears to be a reliable and acceptable strategy for increasing the convenience and reducing the cost of home-based HIV testing [38]. Antenatal counselling sessions should be used as an opportunity to educate men to recognise and help address the mother’s antenatal and postnatal health needs. By establishing a welcoming atmosphere, providers delivering ANC services could also encourage men to attend postnatal visits and engage them in infant health promotion. Male partner support for infant feeding choices can contribute to successful PMTCT through exclusive breastfeeding practices [6,7]—for example, male partners can share in the feeding work by using expressed milk, thereby relieving the mother of the feeding burden.
2. **Expand MNCH service provision to link more effectively to the specific health needs of male clients.** At the facility level, MNCH or ANC staff ought to be engaged as agents in transforming facilities into gender-sensitive spaces, where women and men are welcomed as clients. Staff members thus need to be trained on the distinct health needs of women and men and on appropriate referral pathways. Male-specific health needs may include, for example, medical male circumcision and management of erectile dysfunction. Staff members need to be equipped to recognise and sensitively discuss with clients how gender-inequitable beliefs and behaviours adversely affect health. Stationery used in health facilities could be modified to collect information pertinent to MPI, like marriage and cohabitation status and history of intimate partner violence.
  Attendance of men and couples at services will, however, place additional demands on providers. If national plans and guidelines are as detailed as possible and include realistic costs, facility administrators will be better supported to ensure appropriate staff allocation, monitoring, and remuneration. While MPI in MNCH service provision may carry extra costs, it could ultimately save money. For example, improved retention of women in ANC services would reduce the need for time-intensive tracing and follow-up.
3. **Encourage men to challenge harmful gender-inequitable beliefs and behaviours.** The ability to attend ANC sessions depends on a variety of factors [39], and even men who do not attend ANC sessions have been reported to be involved in pregnancy in a range of different ways, such as providing both emotional and instrumental support to their partners [40]. Developing interventions to improve MPI in a range of pregnancy- and infant-related activities, rather than solely focussing on male attendance at ANC sessions, may thus be beneficial.
  MPI interventions that have been successful in many parts of Africa are those that shift the burden of requesting male participation from women to the health system itself [41]. The barriers preventing men’s involvement can partially be addressed by changes in the system, but coimplementation with community-based interventions is likely to be more effective. Interventions such as the “One Man Can” campaign focus on shifting harmful gender norms and have been shown to increase men’s uptake of HIV counselling and testing [42].
  Therefore, we recommend that health departments and facilities partner with civil society organisations to deliver complementary community engagement efforts. These efforts ought to educate men about MNCH services and transform community-wide gender norms that discourage men from accessing health services. Developing the perception of antenatal couples HIV counselling and testing as a routine service may minimise men’s concerns that attending antenatal clinics is stigmatising and “unmanly” [20]. Interventions involving peer education, social marketing, and endorsements by influential community members may be effective in causing men and women to question and contest rigid inequitable gender norms, or, at least, to disregard them [34]. In relation to MNCH, overturning the attitude that reproductive health is a woman’s responsibility could improve men’s participation in MNCH services. Improving workplace norms to make it easier for men to attend clinics during working hours could be achieved by introducing paternity leave for both pre- and postdelivery support of mothers.

## Challenges That Lie Ahead

Challenges have been identified in delivering clinic-based couples HIV counselling and testing in isolation of other strategies and will need to be addressed in the recommended policy adjustment. Possibly the most significant challenge is that participation rates of male partners in couples counselling and testing interventions have been very low [11–15]. Low uptake is unsurprising given the institutional and social barriers to men’s attendance of MNCH services and implies that simply expanding services to include men may generate only modest improvements in their engagement.

The capacity of health systems to comply with policy documents and national plans also presents a major challenge. Although national and provincial documents that include progressive language and details like how specific tasks ought to be allocated between health professionals will be an important step forward, effective financial cost calculation, implementation, and monitoring will also be essential. Harmful gender norms are present in communities but are similarly prevalent amongst health system staff, including health managers, thereby presenting the need to shift gender norms within the system as a necessary step to enhancing capacity.

The nature of relationships between women and their partners has an important influence on the nature and extent of MPI [20]. Nonmarried or non-cohabiting partnership status acts as a barrier to women disclosing their HIV status to their partners [43], while it is reported that cohabiting and being in a stable relationship facilitates MPI [44,45]. In this article, the words “couple” and “partner” have been used to refer to biological parents who have conceived a child together. These terms may imply a marriage or stable affiliation-based relationship. The reality in South Africa is, however, much more complex. The majority of children do not live with their biological father—the proportion of children whose fathers were absent and living increased between 1996 and 2009 from 42% to 48% [46]. The success of MPI interventions may thus hinge on engaging effectively with the varied nature of partnerships between coparents. Men who have fathered a child and are no longer involved, or are “absent fathers,” have admitted that poverty prohibits them from being involved, since they identify the role of father with the role of being a financial provider [47]. A potential solution may be to use community-based interventions to shift norms around provision from focusing solely on financial provision to include care giving.

## Conclusions

Improving guidelines is an important step to improving MPI, as is effective implementation and monitoring of these guidelines. We have attempted to give an overview of some of the promising evidence and strategies. The nature of relationships and family structures in South Africa and the capacity of health systems present significant challenges and would require innovative solutions to engaging men effectively. The same harmful gender norms that prevent men from participating in couples testing or antenatal care keep men from looking after their own health needs. Engaging men in sexual and reproductive health and MNCH services may be difficult but cannot be ignored in order to harness the benefits for the health needs of mothers, infants, and fathers.

## Abbreviations:

ANC: ante-natal care
MNCH: maternal, neonatal, and child health
MPI: male partner involvement
NAF: National Action Framework
NSP: National Strategic Plan on HIV, STIs and TB
PMTCT: preventing mother-to-child transmission
UNAIDS: the Joint United Nations Programme on HIV and AIDS
