# Correction: Improving Men’s Participation in Preventing Mother-to-Child Transmission of HIV as a Maternal, Neonatal, and Child Health Priority in South Africa

**DOI:** 10.1371/journal.pmed.1001836

**Published:** 2015-05-11

**Authors:** Wessel van den Berg, Kirsty Brittain, Gareth Mercer, Dean Peacock, Kathryn Stinson, Hanna Janson, Vuyiseka Dubula

An affiliation for the last author is not indicated. Vuyiseka Dubula is also affiliated with: University of KwaZulu-Natal, Durban, South Africa.

## References

[1] van den Berg W, Brittain K, Mercer G, Peacock D, Stinson K, et al (2015) Improving Men’s Participation in Preventing Mother-to-Child Transmission of HIV as a Maternal, Neonatal, and Child Health Priority in South Africa. PLoS Med 12(4): e1001811 doi:10.1371/journal.pmed.1001811 25849433 10.1371/journal.pmed.1001811 PMC4388663

